# Over-Expression of *LcPDS*, *LcZDS*, and *LcCRTISO*, Genes From Wolfberry for Carotenoid Biosynthesis, Enhanced Carotenoid Accumulation, and Salt Tolerance in Tobacco

**DOI:** 10.3389/fpls.2020.00119

**Published:** 2020-02-26

**Authors:** Chen Li, Jing Ji, Gang Wang, Zhaodi Li, Yurong Wang, Yajun Fan

**Affiliations:** ^1^ School of Environmental Science and Engineering, Tianjin University, Tianjin, China; ^2^ Division of Biological Sciences, University of California, San Diego, San Diego, CA, United States

**Keywords:** carotenoids, desaturase, isomerase, salt stress, wolfberry

## Abstract

It is of great importance to combine stress tolerance and plant quality for breeding research. In this study, the role of phytoene desaturase (PDS), ζ-carotene desaturase (ZDS) and carotene isomerase (CRTISO) in the carotenoid biosynthesis are correlated and compared. The three genes were derived from *Lycium chinenses* and involved in the desaturation of tetraterpene. Their over-expression significantly increased carotenoid accumulation and enhanced photosynthesis and salt tolerance in transgenic tobacco. Up-regulation of almost all the genes involved in the carotenoid biosynthesis pathway and only significant down-regulation of lycopene ε-cyclase (ε-LCY) gene were detected in those transgenic plants. Under salt stress, proline content, and activities of catalase (CAT), peroxidase (POD) and superoxide dismutase (SOD) were significantly increased, whereas malonaldehyde (MDA) and hydrogen peroxide (H_2_O_2_) accumulated less in the transgenic plants. The genes encoding ascorbate peroxidase (APX), CAT, POD, SOD, and pyrroline-5-carboxylate reductase (P5CR) were shown to responsive up-regulated significantly under the salt stress in the transgenic plants. This study indicated that *LcPDS*, *LcZDS*, and *LcCRTISO* have the potential to improve carotenoid content and salt tolerance in higher plant breeding.

## Introduction

Carotenoids are a series of organic pigments, which spread widely in plants, animals, algae, some special fungi, and bacteria. In higher plants, carotenoids serve as accessory pigments in the photosystem assembly and play essential roles in light-harvesting and photoprotection. Carotenoids are mainly 40-carbon tetraterpene, which consists of eight isoprene units (Goodwin, 1971). In plants, carotenoids are synthesized in plastids from isoprenoid precursors through reactions under the catalyzation by nucleus-encoded enzymes. The condensation of isopentenyl diphosphate (IPP) and dimethylallyl diphosphate (DMAPP) molecules is accomplished to form 15-*cis*-phytoene, which mediated by isopentenyl pyrophosphate isomerase (IPI), geranylgeranyl pyrophosphate synthase (GGPS), and phytoene synthase (PSY) (Fraser et al., 2000; Yang et al., 2016). Subsequently, to transform phytoene into lycopene, plants employ two desaturases, phytoene desaturase (PDS), forming 11 and 11' *cis* double bond, and ζ-carotene desaturase (ZDS), forming 7 and 7' *cis* double bond (Bartley et al., 1991; Araya-Garay et al., 2014). Meanwhile, to fulfill the geometrical requirements of the desaturases, carotene isomerase (CRTISO) is employed to transform 9,15,9'-*tricis*-ζ-carotene into 9,9'-*dicis*-ζ-carotene, 7,9,9'-*tricis*-neurosporene into 9-*cis*-neurosporene and 7,9-*dicis*-lycopene into all-*trans*-lycopene (Han et al., 2008; Yu et al., 2011). The reformed conjugated system, which contained eleven C-C double bonds in the tetraterpene skeleton, makes the molecules easier to absorb light energy and become excited (Hashimoto et al., 2018), and it is important for the functioning of carotenoids. Finally, lycopene is transformed into its varieties containing cycle-, hydroxy-, and epoxide-groups, such as β-carotene, violaxanthin, zeaxanthin, lutein, and neoxanthin, etc. under the catalyzation of lycopene β-cyclase (β-LCY), ε-cyclase (ε-LCY), and β-carotene hydroxylase (β-CHY) (Bouvier et al., 2000; Kim and DellaPenna, 2006; Zhu et al., 2008).

As essential secondary metabolites, carotenoids have been implicated in a large number of biological functions of plant growth and development. In photosystem II (PS II), carotenoids transfer energy to the reaction center (Bassi et al., 1993; Amarnath et al., 2016) and protect chlorophyll from excessive energy by sequestering single oxygen (Di Mascio et al., 1989; Ramel et al., 2012; Ruban, 2016; Leuenberger et al., 2017). Meanwhile, carotenoids play roles in protecting plants from oxidative damages as effective antioxidants (Truscott, 1990). When fruits are ripening, carotenoids are involved in the synthesis of aromatic components (Winterhalter and Rouseff, 2001). As pigments, carotenoids color fruits brightly and attract herbivore to graze (Veen, 2005). The conjugated system makes carotenoids essential for plant growth and development, especially in transferring energy in photosynthesis (Bassi et al., 1993), and the desaturation process is the key procedure to introduce conjugated structure into molecules. The dysfunction of the genes involved in the desaturation process leads to the deficiency of carotenoid accumulation and the death of the plants (Wang M. et al., 2009). The unique phenomenon of pale leaves and lethal phenotype of the plants, which caused by inhibiting the desaturation process, have been usually used to evaluate the efficiency of genetic modification technology (Kaur et al., 2018). Meanwhile, carotenoids are also used as nutrient supplements (Leelakanok et al., 2018) and chemotherapy reagents (Noto et al., 1996). Genes involved in the carotenoid biosynthesis have been isolated and identified for enzymatic activities analysis (Norris et al., 1995; Liu et al., 2013; Brausemann et al., 2017), nutritional enhancements of food (Wurtzel et al., 2003) and genetical modification technology tools (Velasquez et al., 2009; Kaur et al., 2018). Recently, some studies expounded that the over-expression increased the carotenoid content and improved the tolerance to abiotic stresses. Over-expression of *IbZDS* (Li et al., 2017) and *Ibβ-LCY* (Kim et al., 2014) genes enhanced transgenic plants to salt stress tolerance and plants over-expressing *Lcϵ-LCY* enhanced tolerance to chilling stress (Chen et al., 2015).

Wolfberry (*Lycium chinenses*) is a woody plant that spreads in semi-drought areas of China and shows considerable tolerance to abiotic stresses (Chin et al., 2003). The red fruits of wolfberry, which contain a high level of carotenoids, are treated as healthy food and widely used in traditional Chinese medicine and considered as promising resources of candidate genes for genetic modification to enhance the accumulation of carotenoids and tolerance to abiotic stresses of plants. In the present study, genes encoding PDS, ZDS, and CRTISO were derived from red fruits of wolfberry. Their over-expression increased carotenoid content and enhanced salt tolerance significantly in transgenic tobacco.

## Materials and Methods

### Cloning and Sequence Analysis of *LcPDS*, *LcZDS*, and *LcCRTISO*

*L. chinense* used in this study were grown in the greenhouse of the Institute of Genetic Engineering, Tianjin University (Tianjin, China). The red ripen fruits were collected and stored in liquid nitrogen (**Figure S7**). *LcPDS* (Accession No. KJ143993.1), *LcZDS* (Accession No. KJ174516.1), and *LcCRTISO* (Accession No. KJ700839.1) were cloned from the red ripen fruit of wolfberry. The bio-information of *LcPDS*, *LcZDS*, and *LcCRTISO* was analyzed. The open reading frame (ORF) of the derived genes was predicted and translated by using the ExPASy translation tool (https://web.expasy.org/translate/). The parameters of the translated proteins, which refers to “pI,” “molecular weight,” and “length of the ORF,” were predicted with the ExPASy PrtoParam tool (http://web.expasy.org/protparam/). The multiple sequence alignments were finished with DNAMAN software. The phylogenic trees of the translated proteins were constructed with MEGA7software (Felsenstein, 1985; Saitou and Nei, 1987; Nei and Kumar, 2000; Kumar et al., 2016). Information of the species and genes involved in the analysis were listed in **Table S1**. Analysis of 3D protein structures was done with SWISS-MODEL Workspace (https://swissmodel.expasy.org/). All the analysis was done with default parameters. Plasmids pET-28a-*LcPDS* and pET-28a-*LcZDS* were constructed and transformed into *Escherichia coli* for expression analysis. Plasmid pACCRT-EB, which carries geranylgeranyl diphosphate synthase (*crtE*) and 15-*cis*-phytoene synthase (*crtB*), was co-transformed with pET-28a-*LcPDS* into *E. coli* for the enzymatic analysis of *LcPDS*. Plasmid pACCRT-EBP, which carries *crtE*, *crtB*, and 15-*cis*-phytoene desaturase (*crtP*), was co-transformed with pET-28a-*LcZDS* into *E. coli* for enzymatic analysis of *LcZDS*.

### Transgenic Constructs and Generation of Transgenic Tobacco

Binary vector pCambia2300 was used in this study for the over-expression of *LcPDS*, *LcZDS*, and *LcCRTISO* under the control of *CaMV 35S* promoter. After a 3-day vernalization at 4°C, surface-sterilized seeds were sown on growth medium containing half-strength Murashige and Skoog (MS) basal salts, 1.5% (w/v) sucrose, pH 5.75 and 0.7% (w/v) agar, and transgenic tobaccos were obtained by the leaf disk method *via Agrobacterium tumefaciens* (Horsch et al., 1985). Phenotypes of transgenic plants were examined in the T2 generation and confirmed in the following T3, T4, and T5 generation with homozygous seeds. Transgenic tobacco containing an empty vector was set as control (CK). Primers used in this section were listed as **Tables S2** and **S3**.

### Carotenoid Extraction and High-Performance Liquid Chromatography Analysis

Leaf samples were collected and stored in liquid nitrogen, and freeze drier was employed to achieve dried samples; 0.1g sample was grounded into a fine powder. In order to transform carotenoids from esterified to free form, the sample was saponified by being resuspended with 10 ml methanol containing 6% (w/v) potassium hydroxide (KOH) and heat for 20 min at 60°C. The saponified sample was cooled to room temperature and 20 ml ether was added in, and after the partitioning, the upper layer was collected and dried with nitrogen.

For high-performance liquid chromatography (HPLC) analysis, the dried sample was dissolved in 50 μl acetone. Twenty microliters of the aliquot was injected and separated on a Nucleosil 100-3 C18, 250×4.6 mM (MN, Germany) column at 32°C, with acetonitrile/methanol/isopropanol (85:10:5, v/v) as mobile phase at a 1 ml·min^−1^ flow rate. Authentic reference compounds of carotenoids (**Table S4**) were purchased from Sigma-Aldrich. Twenty microliters of 20 μg·ml^−1^ each standard reference compound was loaded and the spectrum was applied for the identification and quantitation of the samples by comparing the retention time and peak area (**Figure S9**). Samples were monitored with a Kontron DAD 440 photodiode array detector. In order to protect the samples from being oxidized, solvents were saturated with nitrogen gas and operations were finished under nitrogen atmospheres.

### Salt Stress Treatments and Physiological Analysis

#### Seed Germination Rates

For analysis of the seed germination rates, 100 sterilized seeds of each genotype were sown on the culturing medium containing 200 mM NaCl. Germination was defined as the obvious emergence of the radicle through the seed coat and percentage was counted as the indicated times.

#### Salt Treatments of the Young Seedlings

In order to analyze the response of young transgenic seedlings to salt stress, tobacco seeds were germinated on normal 1/2 MS medium and then transferred to medium supplemented with 200 mM NaCl for 14 days. Seedlings were photographed and the lengths of primary roots were measured. For salt tolerance analysis, seedlings of 14 day after germination (DAG) grown in soil were treated with 200 mM NaCl. After 14 days' salt treatment, survival rates were calculated and physiological analysis was applied. Relative water content was determined according to the methods described by Barrs (Barrs and Weatherley, 1962). Leaf disks were collected using a leaf puncher on 12:00–14:00. Leaf disks were weighed and placed floating on the water surface for 6 h for turgidity in a 4°C refrigerator. Turgid samples were then weighed and dried in an 80°C oven for 12 h to get the dry weight.

#### Assay of Photosynthetic Process and Measurements of Chlorophyll Content

After 14 days' 200 mM NaCl treatments, the photosynthesis rate (Pn) and photochemical efficiency of photosystem II (Fv/Fm) analysis were performed by using LI 6400 portable apparatus (LI-COR, USA) with a pulse amplitude modulation (PAM-2000) portable fluorometer according to the manufacturer's instructions. The time of the measurement was from 11:30 to 14:00 and the dark-adaption was applied by placing plants in boxes covered with aluminum foil for 30 min. Then, chlorophyll was extracted and measured spectrophotometrically according to methods described by Wintermans (Wintermans and De Mots, 1965).

#### Assay of Oxidative Stress Responses

The fresh leaves of the seedlings treated with 200 mM NaCl for 14 days were collected, contents of hydrogen peroxide (H_2_O_2_) (Zhang and Jiang, 2001) and malonaldehyde (MDA) (Mark Hodges et al., 1999) were assayed as the indicators of oxidation as described. Activities of CAT (EC 1.11.1.6), POD (EC 1.11.1.7), and SOD (EC 1.15.1.1), which are responsible for decomposing peroxide and superoxide, were measured spectrophotometrically according to former methods (Donahue et al., 1997). The accumulation of proline, which was beneficial for plants to acclimatize multiple abiotic stresses, was estimated as previously reported (Bates et al., 1973).

### Ribonucleic Acid Extraction and Gene Expression Analysis

The total RNA of leaves was extracted by RNeasy plant mini kit (QIAGEN) following the manufacturer's instruction. Gene expression level analysis was performed by quantitative real-time PCR using a model real-time PCR system (Agilent Mx3000P) with SYBR Premix Ex Taq™ (Takara). Specific primers used in quantitative real-time (qRT)-PCR were listed in **Table S3**. Relative gene expression levels were normalized to reference gene *NtActin*. Real-time PCR data were calculated with the comparative Ct method. Three biological replicates were performed for each amplification and each PCR was performed in triplicates independently.

### Statistical Analysis

All data were obtained for at three independent experiments and one-way analysis of variance (ANOVAs) was performed with Prism 7 software (GraphPad Software Inc, USA) to compare the differences among groups. The results were presented as means ± standard deviation (SD). P values of < 0.05 (*), < 0.01 (**) and < 0.001 (***) were considered to be significant statistically.

## Results

### Sequence Analysis of *LcPDS*, *LcZDS*, and *LcCRTISO*

The comparing gene information of *LcPDS*, *LcZDS*, and *LcCRTISO* was listed in **Table S5**. The translated protein sequence alignments (**Figures S1**–**S3** ) showed that the *LcPDS*, *LcZDS*, and *LcCRTISO* coding protein had high identities of amino acid sequence with predicted proteins of *Arabidopsis thaliana* (81.49, 79.93, and 88.03%), *Capsicum annuum* (95.19, 94.05, and 94.54%), *Glycine max* (80.18, 81.75, and 86.27%), *Nicotiana tabacum* (95.19, 94.9, and 94.35%), *Oryza sativa* (84.25, 78.55, and 83.05%), and *Solanum lycopersicum* (94.33, 98.66, and 94.7%), respectively. The 3D protein structures were analyzed with SWISS-MODEL Workspace and shown in **Figure S4**. Conserved desaturase domain and binding domains for FAD were predicted in *LcPDS* and *LcZDS* coding proteins. No biologically relevant ligands combining site was identified in *LcCRTISO* coding protein, as CRTISO protein from other plants done. The CRTISO were identified as gamma-carotene desaturase. The phylogenic trees demonstrated that *LcPDS*, *LcZDS*, and *LcCRTISO* coding protein were closely related to the corresponding genes in referenced species (**Figure S5**). A brief color complementation experiment (**Figure S8**) showed that expression of *LcPDS* or *LcZDS* was able to finish the catalytic function and change the color of the *E. coli* dots.

### Over-Expression of *LcPDS*, *LcZDS*, and *LcCRTISO* Improved Tobaccos Carotenoid Accumulation

**Figure 1:**
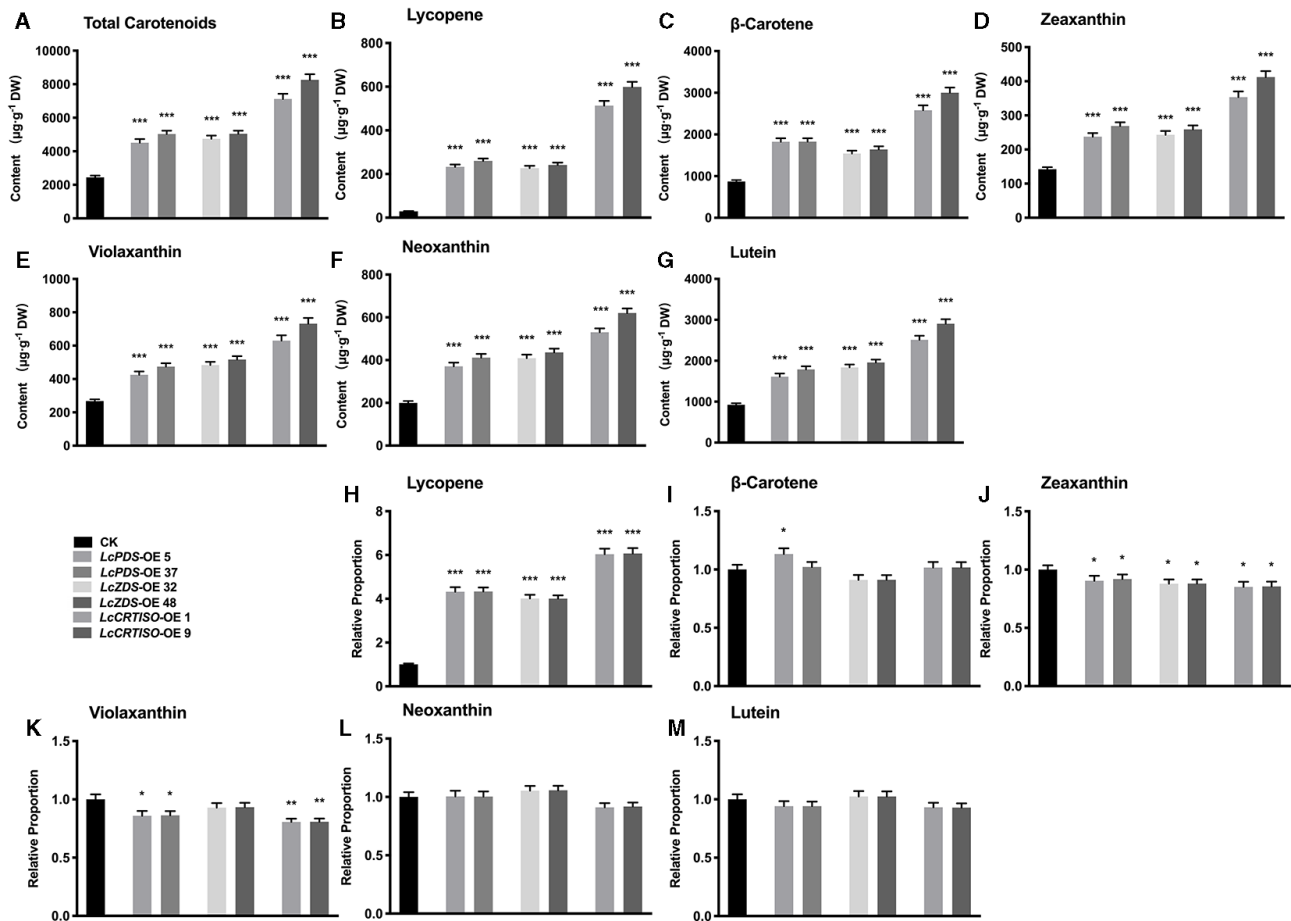
Analysis of carotenoids in *LcPDS*, *LcZDS*, and *LcCRTISO* over-expressing tobacco lines with HPLC. Content of **(A)** total carotenoids, **(B)** lycopene, **(C)** β-carotene, **(D)** zeaxanthin, **(E)** violaxanthin, **(F)** neoxanthin, and **(G)** lutein. Relative proportion of **(H)** lycopene, **(I)** β-carotene, **(J)** zeaxanthin, **(K)** violaxanthin, **(L)** neoxanthin **(M)** lutein were analyzed in freeze-dried samples and shown as figure. Data were obtained from three independent experiments and one-way analysis of variance (ANOVAs) was performed. The results were presented as means ± standard deviation (SD). P values of < 0.05 (*), < 0.01 (**), and < 0.001 (***) were considered to be significant statistically.

Independent over-expression lines were obtained for each genotype, and their gene expression level (**Figures S6A–C**) and total carotenoids (**Figures S6D–F**) were detected with homozygous plants. The results showed that the content of carotenoids was correlated with the expression level of exogenous genes. The transgenic lines which showed highest gene expression level and total content in each genotype were selected for further experiments, *LcPDS*-OE 5, *LcPDS*-OE 37, *LcZDS*-OE 32, *LcZDS*-OE 48, *LcCRTISO*-OE 1, and *LcCRTISO*-OE 9, respectively. Compared to the control plants, total carotenoids in *35S:LcPDS*, *35S:LcZDS*, and *35S:LcCRTISO* increased significantly. The content of total carotenoids in *35S:LcCRTISO* was significantly higher than those in *35S:LcPDS* and *35S:LcZDS*, while no significant difference between *35S:LcPDS* and *35S:LcZDS* was observed. The components of carotenoids were then examined by HPLC and found content for each detected pigment was increased significantly in *35S:LcPDS*, *35S:LcZDS*, and *35S:LcCRTISO* tobaccos. However, the proportions of carotenoid components varied significantly among the three over-expressed tobaccos, showing a significant increase in lycopene and a slight decrease in other components (**Figure 1**).

### Over-Expression of *LcPDS*, *LcZDS*, and *LcCRTISO* Enhanced Tobaccos Salt Tolerance

**Figure 2:**
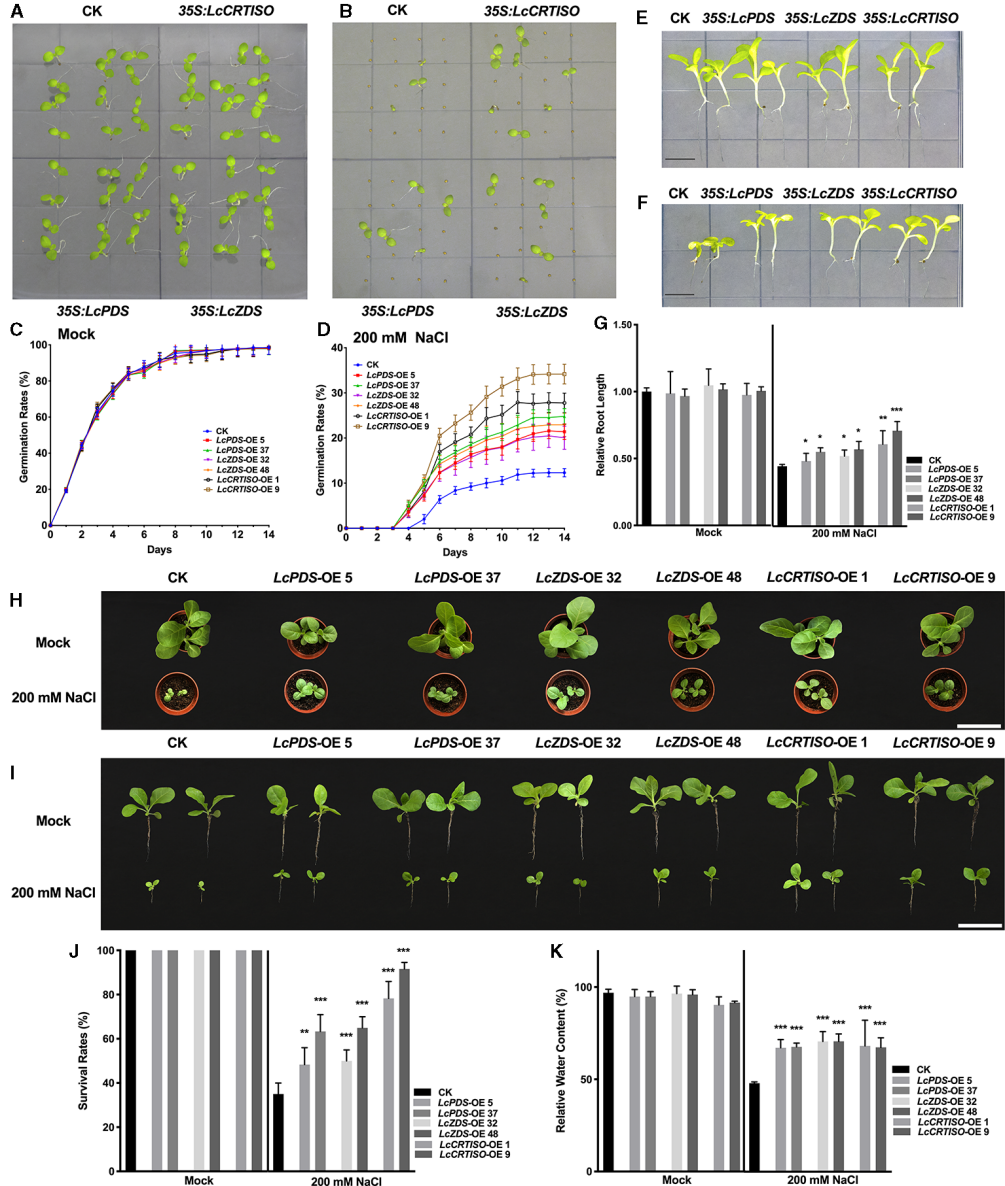
Salt tolerance analysis of *LcPDS*, *LcZDS*, and *LcCRTISO* over-expressing tobacco lines. **(A)** Tobacco seeds germinated in 1/2 Murashige and Skoog (MS) and **(B)** 1/2 MS with 200 mM NaCl of 14 day after germination (DAG) were photographed. Germination rates of seeds were calculated in **(C)** 1/2 MS and **(D)** 1/2 MS with 200 mM NaCl respectively. Seedlings in **(E)** absence and **(F)** presence of salt stress were photographed, and **(G)** the lengths of roots were measured. Scale bars = 1 cm. **(H**, **I)** Phenotypes and **(J)** survival rates of transgenic tobacco lines under salt stress for 14 days. Scale bars = 10 cm. **(K)** Relative water content was measure. Data were obtained from three independent experiments and ANOVAs was performed. The results were presented as means ± SD. P values of < 0.05 (*), < 0.01 (**), and < 0.001 (***) were considered to be significant statistically.

Germination rates of *35S:LcPDS*, *35S:LcZDS*, and *35S:LcCRTISO* tobaccos were examined under salt stress. Vernalized seeds of each genotype were sown on 1/2 MS medium supplemented with 200 mM NaCl, and the germination rates were recorded for 2 weeks (**Figures 2A–D**). Under salt stress, the seed germination was inhibited and delayed, and the three over-expressors showed higher germination rates. From day 3 to day 14, an increasing difference between the over-expression and control plants was observed. The germinating seeds of *35S:LcPDS*, *35S:LcZDS*, and *35S:LcCRTISO* tobaccos were 173.5–201.2, 162.7–186.1, and 225.4–277.3% compared to that of the control plants respectively, which showing obvious higher tolerance to salt stress during seed germination.

**Figure 3:**
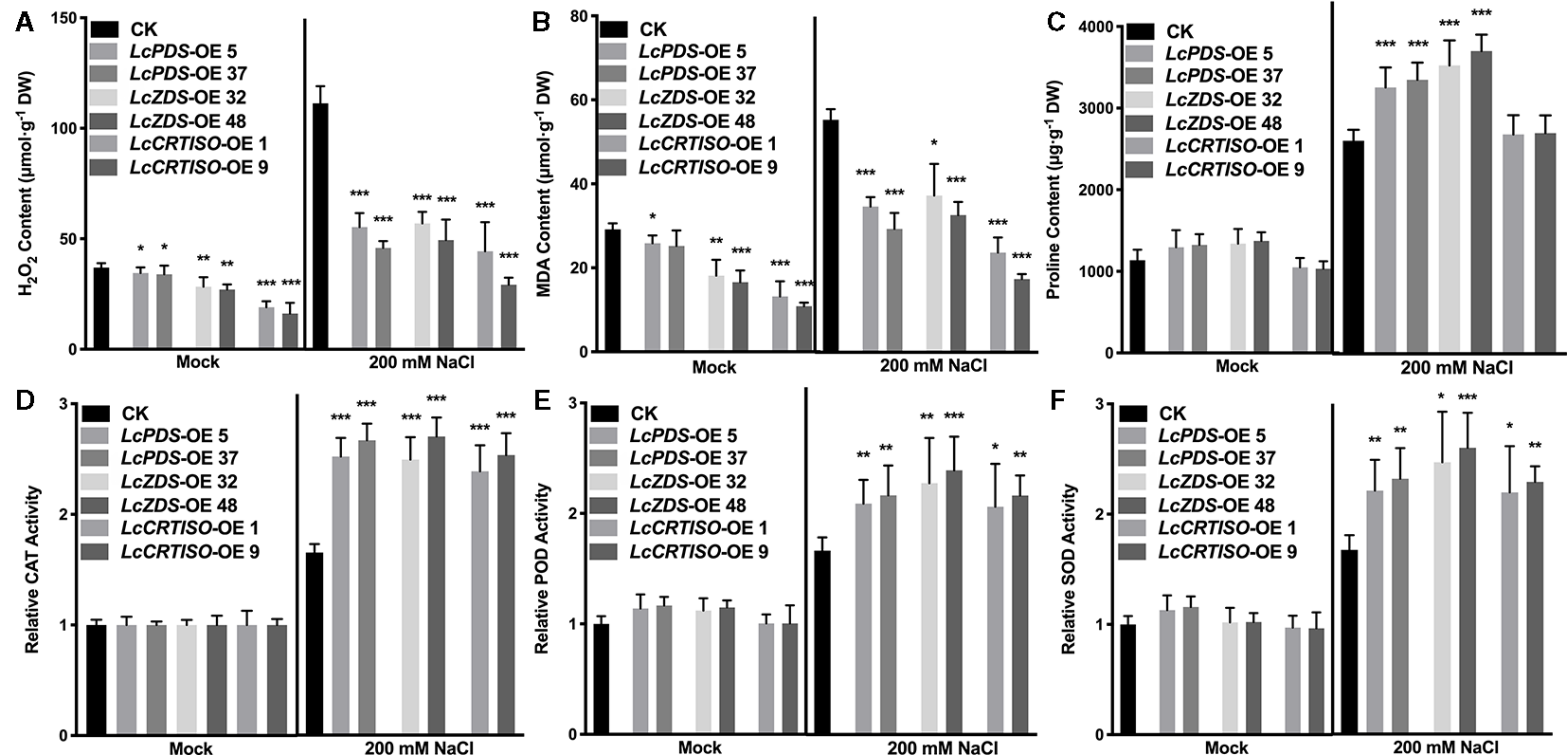
Antioxidative components in *LcPDS*, *LcZDS*, and *LcCRTISO* over-expressing tobacco lines were analyzed. The contents of **(A)** H_2_O_2_, **(B)** malonaldehyde (MDA), and **(C)** proline were assayed, and activities of **(D)** catalase (CAT), **(E)** peroxidase (POD), and **(F)** superoxide dismutase (SOD) were analyzed. Data were obtained from three independent experiments and ANOVAs was performed. The results were presented as means ± SD. P values of < 0.05 (*), < 0.01 (**), and < 0.001 (***) were considered to be significant statistically.

Then, 7-day old young seedlings were transferred to 1/2 MS medium containing 200 mM NaCl and phenotypes were analyzed. After 14 days' treatment, seedlings were photographed and lengths of primary roots were measured (**Figures 2E–G**). Salt stress inhibited the growth of tobaccos and resulted in smaller shoots and shortened primary roots, the root lengths of control plants were reduced by 55.4%, while those of *35S:LcPDS*, *35S:LcZDS*, and *35S:LcCRTISO* tobaccos were reduced by 42.7–50.7, 43.6–50.1, and 29.3–37.5% respectively. The salt tolerance of tobaccos grown in soil was also determined with seven DAG seedlings treated with 200 mM NaCl for every second day. After 21 days' treatment, the survival rates were reduced significantly, and only 35% of the control plants survived (**Figures 2H–J**). Meanwhile, almost 48.3–63, 50–65, and 78.3–91.7% of the plants survived in *35S:LcPDS*, *35S:LcZDS*, and *35S:LcCRTISO* lines, respectively (**Figures 3D–F**). The relative water content (RCW) of the seedlings over-expressing *LcPDS*, *LcZDS*, and *LcCRTISO* were about 67, 70, and 67%, respectively, and at the same time, the RCW of the control plants was only 47.1% (**Figure 2K**).

Further, the contents of H_2_O_2_ and MDA were examined. Compared with control plants, lower contents of H_2_O_2_ and MDA were observed in *LcPDS* (41.2–49.7% and 53.1–62.7%), *LcZDS* (44.4–51.2% and 59.1–67.5%), and *LcCRTISO* (26.3–39.9% and 46%) over-expression tobaccos (**Figures 3A, B**). Meanwhile, under salt stress, activities of CAT, POD, and SOD showed a dramatically increase of *35S:LcPDS* (239.2–263.0, 145.3–152.7, and 160.6–172.1%), *35S:LcZDS* (234.8–268.5, 178.2–192.4, and 215.2–234.5%), and *35S:LcCRTISO* (217.9–241.3, 163.5–179.9, and 181.8–196.8%) tobaccos in comparison with the control plants, respectively (**Figures 3D–F**).

### Over-Expression of *LcPDS*, *LcZDS*, and *LcCRTISO* Improved Tobaccos’ Photosynthesis

**Figure 4:**
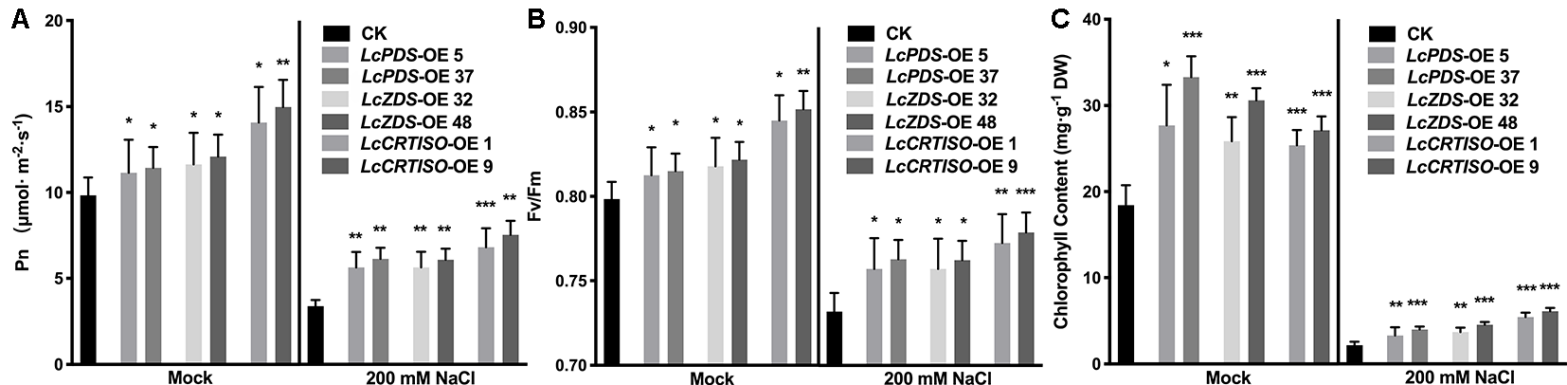
Photosynthetic analysis in *LcPDS*, *LcZDS*, and *LcCRTISO* over-expressing tobacco lines under salt stress were assayed. **(A)** Net photosynthesis rates, **(B)** maximum quantum efficiency of PSII, and **(C)** chlorophyll content in the leaves were measured. Data were obtained from three independent experiments and ANOVAs was performed. The results were presented as means ± SD. P values of < 0.05 (*), < 0.01 (**), and < 0.001 (***) were considered to be significant statistically.

Photosynthetic rate (Pn) and maximal photochemical efficiency of PS II (Fv/Fm) were measured to evaluate *LcPDS*, *LcZDS*, and *LcCRTISO* over-expression effecting the tobacco photosynthesis. Both parameters were much higher under mock and salt stress treatments in *35S:LcPDS*, *35S:LcZDS*, and *35S:LcCRTISO* tobaccos, which implied a photosynthesis improvement (**Figure 4**). Under salt stress, the Pn and Fv/Fm in over-expressors were almost 1.67–2.32 and 1.03–1.06 folds of the control plants (**Figures 4A, B**). Meanwhile, the analytical data showed that the chlorophyll content of the three over-expressed plants had an increasing trend, which was 1.38–1.81 and 1.52–2.81 folds of control plants under mock and salt stress treatments, respectively (**Figure 4C**).

### Correlation Analysis of Salt Tolerance and Carotenoid Content

**Figure 5:**
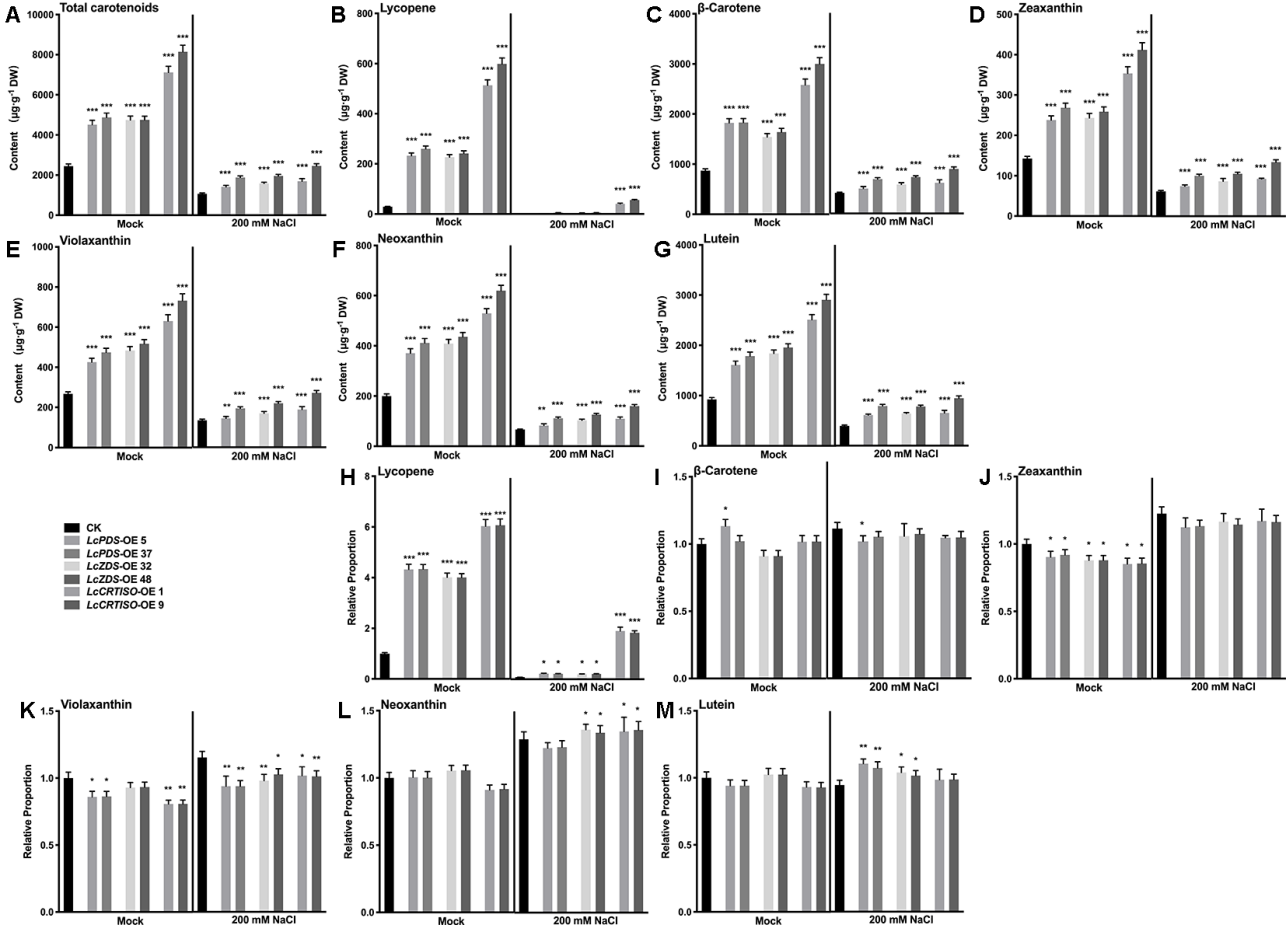
Analysis of carotenoids in *LcPDS*, *LcZDS*, and *LcCRTISO* over-expressing tobacco lines under salt stress. Content of **(A)** total carotenoids, **(B)** lycopene, **(C)** β-carotene, **(D)** zeaxanthin, **(E)** violaxanthin, **(F)** neoxanthin, and **(G)** lutein. Relative proportion of **(H)** lycopene, **(I)** β-carotene, **(J)** zeaxanthin, **(K)** violaxanthin, **(L)** neoxanthin **(M)** lutein were analyzed in freeze-dried samples and shown as figure. Data were obtained from three independent experiments and ANOVAs was performed. The results were presented as means ± SD. P values of < 0.05 (*), < 0.01 (**), and < 0.001 (***) were considered to be significant statistically.

In order to confirm the relationship between the accumulated carotenoids and the enhanced salt tolerance in *35S:LcPDS*, *35S:LcZDS*, and *35S:LcCRTISO* tobaccos, the contents of total carotenoids and main components were detected. Under salt stress, the contents of total carotenoids and each detected component decreased significantly in each transgenic tobacco, lycopene in particular decreased significantly (**Figure 5**). It was speculated that the consumption of carotenoids was related to the salt tolerance of plants. In addition, the proportions of various carotenoids remained unchanged or increased slightly, while lycopene decreased significantly. It was suggested that lycopene was more important than other carotenoid components in the process of transgenic tobacco exposure to salt stress (**Figure 5**). Furthermore, compared with *35S:LcPDS* and *35S:LcZDS* tobaccos, *35S:LcCRTISO* left more lycopene after salt stress, suggesting that there may be enough lycopene for consumption and thus contribute to better performance (**Figure 5**).

### Expression Analysis of Carotenoid Biosynthesis Pathway and Responsive Genes

**Figure 6:**
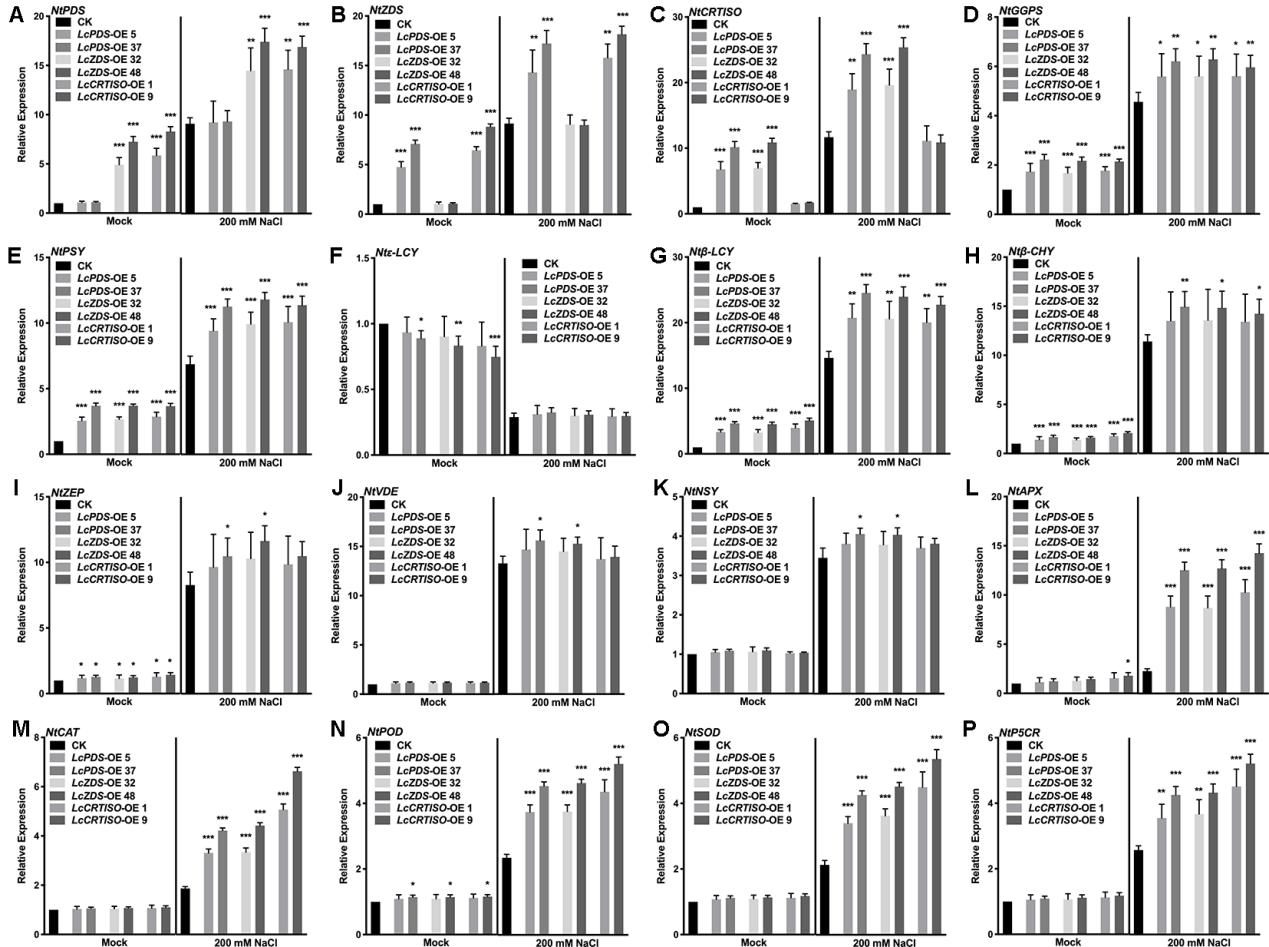
Expression analysis of genes involved in the carotenoid biosynthesis and the salt stress-response in *LcPDS*, *LcZDS*, and *LcCRTISO* over-expressing tobacco lines under salt stress. Gene expression level of **(A)** *NtPDS*, **(B)** *NtZDS*, **(C)** *NtCRTISO*, **(D)** *NtGGPS*, **(E)** *NtPSY*, **(F)** *Ntϵ*-*LCY*, **(G)** *Ntβ-LCY*, **(H)** *Ntβ*-*CHY*, **(I)** *NtZEP*, **(J)** *NtVDE*, **(K)** *NtNSY*, **(L)** *NtAPX*, **(M)** *NtCAT*, **(N)** *NtPOD*, **(O)** *NtSOD*, and **(P)** *NtP5CR* were measured by quantitative real-time (qRT)-PCR, and *NtActin* was set as control. Data were obtained from three independent experiments and ANOVAs was performed. The results were presented as means ± SD. P values of < 0.05 (*), < 0.01 (**), and < 0.001 (***) were considered to be significant statistically.

The expression level of carotenoid biosynthesis-related genes in tobaccos was detected to investigate the regulatory mechanisms of carotenoid biosynthesis involved in the salt stress tolerance. *NtGGPS*, *NtPSY*, *Ntβ-LCY*, *Ntβ-CHY*, *NtZEP*, *NtVDE*, and *NtNSY* up-regulated significantly in transgenic tobacco lines over-expressing *LcPDS*, *LcZDS*, and *LcCRTISO* respectively, while *Ntϵ-LCY* down-regulated at the same time. *NtPSY*, *Ntβ-LCY*, *Ntβ-CHY*, *NtZEP*, *NtVDE*, and *NtNSY* were induced to up-regulate and *Ntϵ-LCY* down-regulate under 200 mM NaCl stress. Salt stress-responsive genes encoding APX, CAT, POD, SOD, and P5CR up-regulated significantly in the transgenic plants under 200 mM NaCl stress compared with the control group. In *35S:LcPDS*, *35S:LcZDS*, and *35S:LcCRTISO* transgenic tobaccos, respectively, their counterpart genes of *NtPDS*, *NtZDS*, and *NtCRTISO* showed no significant difference on expression level than that in control plants (**Figure 6**).

## Discussion

The carotenoid biosynthesis pathway showed high complexity and involved in various metabolic processes, such as photosynthesis and hormonal adjustments. The over-expression of exogenous *LcPDS*, *LcZDS*, and *LcCRTISO* was found to have a significant impact on the homeostatic state of the carotenoid biosynthesis pathway in the transgenic tobacco and considered to contribute to the change of the phenotype.

In transgenic tobacco over-expressing *LcPDS*, *LcZDS*, or *LcCRTISO*, an increase of carotenoid content in tobacco leaves and the change in the ratio among the various components of carotenoids were determined. The results showed that the content and the ratio of carotenoids in transgenic leaves changed significantly. The carotenoid in leaves was concerned to be conserved in leaves and the over-expression of exogenous genes affected neither the content nor the ratio of the carotenoids (Bartley et al., 1994; Latari et al., 2015). Busch et al. (2002) found that the over-expression of *NtPSY* significantly accelerated the biosynthesis of carotenoids and accumulated extra carotenoids in tobacco leaves. In this study, significant enhancement of carotenoid accumulation in transgenic tobacco was determined, and it was considered that the effects of over-expressed exogenous genes on the carotenoid biosynthesis were influenced by the relationship between the donor and acceptor of the genes.

The research did by Busch et al. (2002) demonstrated that the chlorophyll content in tobacco leaves would be influenced by the over-expression of *NtPSY* and the ratio of chlorophyll to carotenoids altered. In order to determine the effect of the over-expression of *L. chinense*-derived genes in tobacco, the chlorophyll to carotenoids mole ratio was analyzed in this study as well, which was calculated by using the average content of carotenoids and chlorophyll shown in **Figures 1A** and **4C**. It showed that extra chlorophyll accumulated in the leaves and the mole ratio of chlorophyll to carotenoids varied significantly among the transgenic lines, 3.64–3.94 in *35S:LcPDS*, 3.23–3.59 in *35S:LcZDS*, 1.95–2.11 in 35S:*LcCRTISO*, and 4.48 in the control group. It was suggested that chlorophyll content, which was correlated to photosynthesis, was involved in the adaption to the impact caused by the over-expression and influenced by the different types of chemical reactions.

Saponification of the samples was applied in preparation of the HPLC analysis. Carotenoids exist as both free and esterified form in plants. Carotenoids in leaves were considered in non-esterified form and proved with studies (Stange, 2016). Recent studies showed that esterified carotenoids can be detected in leafy tissue of genetically modified plants which produce high content of astaxanthin (Harada et al., 2014; Ogawa et al., 2016). Meanwhile, it was also found that esterified carotenoids could also detected in yellowish green or yellow leaves, which contains no esterified carotenoids when the leaves were green (Metlicar et al., 2019). It was suggested that saponification of the leafy samples, which was treated unnecessary for leafy tissues, should be taken into consideration in studies on senescing, aging or stress-tolerating leafy tissues, and on transgenic plants over-expressing genes involved in the carotenoid biosynthesis.

Further, to get more knowledge about the impacts caused by the over-expression of the exogenous gene involved in the carotenoid synthesis pathway in tobacco and the adaptation of tobacco to the impacts, the expression level of each gene involved in the carotenoid biosynthesis in tobacco was determined and analyzed.

It was inferred that PDS, ZDS, and CRTISO, the three enzymes involved in the desaturation process, collaborated to accomplish the desaturation of carotenoid. The up-regulation of any one of desaturation-related genes resulted in the impact of the homeostatic state among them and the expression level of the other two of the three would be up-regulated to adapt this impact.

Meanwhile, the expression levels of *NtGGPS* and *NtPSY* up-regulated significantly in the three over-expressors, which was inferred to fit the acceleration of carotenoid biosynthesis and accumulation of extra carotenoids. Lycopene, as the direct product of the desaturation process, was determined accumulated in the transgenic tobacco leaves. In dealing with the extra lycopene, up-regulation of endogenous *Ntβ-LCY*, *Ntβ-CHY*, and *NtZEP* in transgenic plants was observed. As a result of the regulation, extra accumulated down-stream products were determined. At the same time, a tendency that plants prefer to stabilize the proportion of carotenoid components in the plant was examined as well.

In addition, the expression level of *Ntϵ-LCY* was exceptionally decreased among the detected genes involved in the carotenoid biosynthesis pathway in the three transgenic tobacco. It inferred the preference to β-cyclized products over ε- ones, by regulating the expression level of *Ntβ-LCY* and *Ntϵ-LCY*.

Under salt stress, it showed higher germination rates, higher survival rates, larger seedling with longer roots, more green leaves with higher relative water content in the three over-expressors.

Indicators of oxidative stress were determined to explore the functions and mechanisms in the enhancement. The production of reactive oxygen species (ROS) during plant metabolism is an inevitable result of electron transport systems under normal conditions. ROS accumulated when plants exposed to salt stress and lead to photo-oxidative stress, and further lead to the degradation of photosynthetic pigments and decreased the photosynthetic capacity of plants (Esterbauer and Cheeseman, 1990; Rustérucci et al., 1999; Choudhury et al., 2017). As the indicator of ROS, H_2_O_2_ and MDA content was detected and shown to be lower in the transgenic plants, which suggested that the transgenic plants which contained higher carotenoid content could scavenge more ROS when exposed to salt stress.

Meanwhile, plants employ antioxidative enzymes to detoxify ROS and prevent plants from oxidative damages (Choudhury et al., 2017). Ascorbate peroxidase (APX) catalase (CAT), peroxidase (POD), and superoxide dismutase (SOD), which were reported to be key genes involved in response to salt stress (Tang et al., 2006; Lim et al., 2007; Ahmad et al., 2008; Wang W-B. et al., 2009), were determined and higher antioxidant enzyme activities and up-regulation of ROS detoxifying pathway genes were observed in the three over-expressors than those in control group under salt stress. It was suggested that the three over-expressors which accumulated higher carotenoids showed a higher capacity to employ enzymes detoxifying ROS under salt stress.

In addition, proline was reported to be involved in quenching singlet oxygen and scavenging ROS (Matysik et al., 2002; Hayat et al., 2012). It plays an osmoprotective role in physiological responses, enabling the plants to better tolerate the adverse effects of abiotic stress as well. The expression level pyrroline-5-carboxylate reductase (P5CR), one of the key genes involved in proline biosynthesis (Djilianov et al., 2005; Kaur and Asthir, 2015), was detected up-regulated at higher levels in the three over-expressors than those in the control plants. Meanwhile, higher proline content was determined accumulated more in the three over-expressors (**Figure 3C**) and it was indicated that accumulating more proline was involved in the salt stress enhancement.

The content of carotenoids and chlorophyll significantly decreased when plants exposed to salt stress. The remaining carotenoids and chlorophyll were significantly higher than those in the mock-treated plants. Meanwhile, the photosynthesis system was shown damaged under salt stress. The photosynthesis system of the three over-expressors performed better than the control group, which indicated that extra accumulated carotenoids were beneficial for plants in the salt stress tolerating process. It was supposed that the extra accumulated carotenoids played an important positive role in response to salt stress, and the performance showed dosage dependent on the carotenoid accumulation.

Under salt stress, the endogenous genes involved in the carotenoid synthesis pathway response to salt stress positively in tobacco by up-regulating the expression levels, while the HPLC results showed that the carotenoid content decreased significantly under salt stress conditions. At the same time, it was determined that the enhancement of salt tolerance in tobacco was positively reacted to the accumulation of carotenoids. The presence of large-capacity of carotenoid was considered to be beneficial for plants to tolerate higher tolerance to salt stress. It was supposed that plants tried to synthesize and accumulate more carotenoids under salt stress, but the rate accelerated by the regulation accomplished by the plants was not able to meet the consumption of carotenoids caused by the salt stress. As the result, the up-regulation of carotenoid biosynthesis related genes and the decrease of carotenoid content were observed at the same time.

The content of carotenoids in *35S:LcCRTISO* was determined higher than those in *35S:LcPDS* and *35S:LcZDS*, when exogenous genes expressed at a similar level. As an isomerase, *LcCRTISO* was shown higher efficiency on enhancing the synthesis and accumulation of carotenoids in tobacco leaves, than the two desaturates (**Figure S6**).

When trying to screen tobacco seedlings with higher carotenoid content, it was found difficult to obtain the plants to meet the requirements. The expression levels of the exogenous genes and contents of carotenoids seemed to be limited under some certain levels. Only a small number of transgenic tobacco lines can express exogenous genes and accumulate carotenoids stably at such a high level. Considered the important roles of carotenoid synthesis-related genes and the complicated metabolic processes in plants are controlled by the strict regulation mechanism in plants, the plants expressed the exogenous genes at an excessively high level which was beyond the ability of regulation in plants, would lead the collapse of metabolism and death to the plants.

In conclusion, *LcPDS*, *LcZDS*, and *LcCRTISO* have been isolated from *L. chinense* and characterized in *N. tabacum*. Their over-expression significantly increased the carotenoid content and enhanced salt tolerance in tobacco. The photosynthesis of the over-expressors were enhanced as well. Meanwhile, it was found that the transgenic tobacco plants showed higher resistance to oxidative stress, by up-regulating the expression level and activity of antioxidant enzymes. This study indicated that *LcPDS*, *LcZDS*, and *LcCRTISO* have the potential for improving carotenoid content and enhancing salt tolerance in tobacco and for other higher plant breeding.

## Data Availability Statement

All datasets for this study are included in the article/**Supplementary Material**.

## Author Contributions

JJ, CL, and GW designed most of the experiments and directed the project. CL performed the entire experiments. ZL contributed to the isolation of the genes and the construction of vectors. YW and YF contributed to the experimental design. CL and JJ analyzed the data and wrote the paper. All of the authors agreed on the content of the paper and declare no conflicting interests.

## Funding

This work was supported by the National Natural Science Foundation of China (31271793 and 31271419) and the Key Technologies R & D Program of Tianjin (19YFZCSN00280).

## Conflict of Interest

The authors declare that the research was conducted in the absence of any commercial or financial relationships that could be construed as a potential conflict of interest.

## Abbreviations

APX, ascorbate peroxidase; CaMV, cauliflower mosaic virus; CAT, catalase; CRTISO, carotene *cis-trans* isomerase; DAG, day after germination; GGPS, geranylgeranyl pyrophosphate synthase; H_2_O_2_, hydrogen peroxide; HPLC, high-performance liquid chromatography; IPI, isopentenyl pyrophosphate isomerase; MDA, malonaldehyde; NADPH, reduced form nicotinamide adenine dinucleotide phosphate; NSY, neoxanthin synthase; P5CR, pyrroline-5-carboxylate reductase; PDS, phytoene desaturase; POD, peroxidase; PS II, photosystem II; PSY, phytoene synthase; ROS, reactive oxygen species; SOD, superoxide dismutase; VDE, violaxanthin de-epoxidase; ZDS, ζ-carotene desaturase; ZEP, zeaxanthin epoxidase; β-CHY, β-carotene 3-hydroxylase; β-LCY, lycopene β-cyclase; ε-LCY, lycopene ε-cyclase.
