# Supplementary material for: Over-Expression of LcPDS, LcZDS, and LcCRTISO, Genes From Wolfberry for Carotenoid Biosynthesis, Enhanced Carotenoid Accumulation, and Salt Tolerance in Tobacco
Source: Front Plant Sci. 2020 Feb 26;11:119. doi: 10.3389/fpls.2020.00119 (PMC7054348; doi:10.3389/fpls.2020.00119)
Supplement: Supplementary file 1 [file DataSheet_1.docx]

**Supplementary Figure 1.** Amino acid alignment of LcPDS with other selected corresponding genes were finished with DNAMAN software. α-Helices, η-helices, β-sheets and β-turns were labeled with bars in red, yellow, green and blue, respectively. Signal peptide was labeled with black frame. AtPDS (*Arabidopsis thaliana*, Q07356), CaPDS (*Capsicum* *annuum*, P80093), GmPDS (*Glycine* *max*, P28553), NpPDS (*Narcissus* *pseudonarcissus*, Q40406), NtPDS (*Nicotiana* *tabacum*, A0A1S4CA25), OsPDS (*Oryza* *sativa*, A2XDA1), SlPDS (*Solanum* *lycopersicum*, P28554), TePDS (*Tagetes* *erecta*, A0A2R4G920) and ZmPDS (*Zea* *mays*, P49086) were involved in the amino acid alignment of LcPDS.

**Supplementary Figure 2.** Amino acid alignments of LcZDS with other selected corresponding genes were finished with DNAMAN software. α-Helices, η-helices, β-sheets and β-turns were labeled with bars in red, yellow, green and blue, respectively. Signal peptide was labeled with black frame. AtZDS (*Arabidopsis* *thaliana*, Q38893), CaZDS (*Capsicum* *annuum*, Q9SMJ3), GmZDS (*Glycine* *max*, I1NIT4), NpZDS (*Narcissus* *pseudonarcissus*, O49901), NtZDS (*Nicotiana* *tabacum*, A0A067YEL0), OsZDS (*Oryza* *sativa*, Q0D7W4), SlZDS (*Solanum* *lycopersicum*, Q9SE20), TeZDS (*Tagetes* *erecta*, Q9FV46) and ZmZDS (*Zea* *mays*, Q9ZTP4) were involved in the amino acid alignment of LcZDS.

**Supplementary Figure 3.** Amino acid alignments of LcCRTISO with other selected corresponding genes were finished with DNAMAN software. α-Helices, η-helices, β-sheets and β-turns were labeled with bars in red, yellow, green and blue, respectively. Signal peptide was labeled with black frame. AtCTRISO (*Arabidopsis* *thaliana*, Q9M9Y8), CaCTRISO (*Capsicum* *annuum*, A0A1U8F6R6), CsaCTRISO (*Cucumis* sativus, A0A0A0LBN7), CsiCTRISO (*Citrus* *sinensis*, A0A067G5N7), GmCTRISO (*Glycine* *max*, A0A0R0L3G5), LsCTRISO (*Lactuca* *sativa*, A0A2J6KRW2), NtCTRISO (*Nicotiana* *tabacum*, A0A1S3ZX27), OsCTRISO (*Oryza* *sativa* , B8BL57), SlCTRISO (*Solanum* *lycopersicum*, Q8S4R4), StCTRISO (*Solanum* *tuberosum*, M1CQH9) and VvCRTISO (*Vitis* *vinifera*, A0A438D0F6) were involved in the amino acid alignment of LcCRTISO.

**Supplementary Figure 4.** 3D protein structures predictions of (a) LcPDS, (b) LcZDS and (c) LcCRTISO. The predictions were applied with SWISS-MODEL Workspace supplied by expasy.org. The template used for the 3D prediction of LcPDS and LcZDS was 5mog.1.A, and the one used for LcCRTISO was 4rep.1.A. As described by the website, blue color means higher estimating score while orange means lower.

**Supplementary Figure 5.** The evolutionary history analysis of (a) LcPDS and LcZDS and (b) LcCRTISO were completed inferred using the Neighbor-Joining method and conducted in MEGA7 software. The LcPDS, LcZDS and LcCRTISO were indicated with ■, ▲ and ●, respectively. AtPDS (*Arabidopsis thaliana*, Q07356), CaPDS (*Capsicum* *annuum*, P80093), GmPDS (*Glycine* *max*, P28553), MxPDS (*Myxococcus* *xanthus*, Q1DDH1), NpPDS (*Narcissus* *pseudonarcissus*, Q40406), NtPDS (*Nicotiana* *tabacum*, A0A1S4CA25), OsPDS (*Oryza* *sativa*, A2XDA1), SePDS (*Synechococcus* *elongatus*, P26294), SlPDS (*Solanum* *lycopersicum*, P28554), VvPDS (*Vitis* vinifera, I7FWT3), AtZDS (*Arabidopsis* *thaliana*, Q38893), CaZDS (*Capsicum* *annuum*, Q9SMJ3), GmZDS (*Glycine* *max*, I1NIT4), MxZDS (*Myxococcus* *xanthus*, Q02861), NpZDS (*Narcissus* *pseudonarcissus*, O49901), NtZDS (*Nicotiana* *tabacum*, A0A067YEL0), OsZDS (*Oryza* *sativa*, Q0D7W4), SeZDS (*Synechococcus* *elongatus*, Q31N27), SlZDS (*Solanum* *lycopersicum*, Q9SE20) and VvZDS (*Vitis* *vinifera*, I7EUP4) were introduced for composing evolutionary tree of LcPDS and LcZDS. AtCTRISO (*Arabidopsis* *thaliana*, Q9M9Y8), CaCTRISO (*Capsicum* *annuum*, A0A1U8F6R6), CsaCTRISO (*Cucumis* sativus, A0A0A0LBN7), CsiCTRISO (*Citrus* *sinensis*, A0A067G5N7), EsCRTISO (*Eutrema* salsugineum, V4KEQ2), GmCTRISO (*Glycine* *max*, A0A0R0L3G5), LsCTRISO (*Lactuca* *sativa*, A0A2J6KRW2), NtCTRISO (*Nicotiana* *tabacum*, A0A1S3ZX27), OsCTRISO (*Oryza* *sativa* , B8BL57), SeCTRISO (*Synechococcus* *elongatus*, A0A3G6X1R9), SlCTRISO (*Solanum* *lycopersicum*, Q8S4R4), StCTRISO (*Solanum* *tuberosum*, M1CQH9) and VvCRTISO (*Vitis* *vinifera*, A0A438D0F6) were introduced for composing evolutionary tree of LcCRTISO.

**Supplementary Figure 6.** Over-expression of *LcPDS*, *LcZDS* and *LcCRTISO* enhanced the carotenoids accumulation in transgenic tobaccos. Expression levels of (a) *LcPDS*, (b) *LcZDS* and (c) *LcCRTISO* were measured by qRT-PCR and relative gene expression levels were normalized to reference gene *NtActin*. Total carotenoids content in (d) *LcPDS*, (e) *LcZDS* and (f) *LcCRTISO* over-expressing tobacco lines were examined spectrophotometrically. Data were obtained from three independent experiments and performed as means ± standard deviation (SD).

**Supplementary Figure 7.** Different stages of wolfberry fruits ripening. The genes were derived from the stage (d) the red ripen fruits.

**Supplementary Figure 8.** The color complementation of *LcPDS* and *LcZDS* in engineered *E.coli*. Recombined *E.coli* stains containing plasmid (a) pACCRT-EB, (b) pACCRT-EB and pET-28a-*LcPDS*, (c) pACCRT-EBP (d) pACCRT-EBP and pET-28a-*LcZDS* were shown. The chromatograms of recombined *E.coli* stains containing plasmid (e) pACCRT-EB, (f) pACCRT-EB and pET-28a-*LcPDS*, (g) pACCRT-EBP (h) pACCRT-EBP and pET-28a-*LcZDS* were displayed.

**Supplementary Figure 9.** The chromatogram figures of HPLC analysis. The chromatogram figures of (a) control plants and tobacco over-expressing (b) *LcPDS*, (c) *LcZDS* and (d) *LcCRTISO* growing under normal condition, the figure of (e) control plants and tobacco over-expressing (f) *LcPDS*, (g) *LcZDS* and (h) *LcCRTISO* growing under salt stress were shown. The chromatogram figures of standard samples were shown in (i), and peaks labeled with numbers were standard samples of (1) neoxanthin, (2) violaxanthin, (3) lutein, (4) zeaxanthin, (5) lycopene, (6) neurosporene, (7) ζ-carotene and (8) β-carotene respectively.
