# Supplementary material for: Over-Expression of LcPDS, LcZDS, and LcCRTISO, Genes From Wolfberry for Carotenoid Biosynthesis, Enhanced Carotenoid Accumulation, and Salt Tolerance in Tobacco
Source: Front Plant Sci. 2020 Feb 26;11:119. doi: 10.3389/fpls.2020.00119 (PMC7054348; doi:10.3389/fpls.2020.00119)
Supplement: Supplementary file 11 [file Table_1.docx]

**Supplementary Table 1.** Information of the protein for the sequence analysis.

| **Protein** | **Species** | **Accession number** |
| --- | --- | --- |
| AtPDS | *Arabidopsis thaliana* | Q07356 |
| CaPDS | *Capsicum annuum* | P80093 |
| GmPDS | *Glycine max* | P28553 |
| LcPDS | *Lycium chinense* | A0A0M3L7K1 |
| MxPDS | *Myxococcus xanthus* | Q1DDH1 |
| NpPDS | *Narcissus pseudonarcissus* | Q40406 |
| NtPDS | *Nicotiana tabacum* | A0A1S4CA25 |
| OsPDS | *Oryza sativa* | A2XDA1 |
| SePDS | *Synechococcus elongatus* | P26294 |
| SlPDS | *Solanum lycopersicum* | P28554 |
| TePDS | *Tagetes erecta* | A0A2R4G920 |
| VvPDS | *Vitis vinifera* | I7FWT3 |
| ZmPDS | *Zea mays* | P49086 |
| AtZDS | *Arabidopsis thaliana* | Q38893 |
| CaZDS | *Capsicum annuum* | Q9SMJ3 |
| GmZDS | *Glycine max* | I1NIT4 |
| LcZDS | *Lycium chinense* | A0A0H3V057 |
| MxZDS | *Myxococcus xanthus* | Q02861 |
| NpZDS | *Narcissus pseudonarcissus* | O49901 |
| NtZDS | *Nicotiana tabacum* | A0A067YEL0 |
| OsZDS | *Oryza sativa* | Q0D7W4 |
| SlZDS | *Solanum lycopersicum* | Q9SE20 |
| SeZDS | *Synechococcus elongatus* | Q31N27 |
| TeZDS | *Tagetes erecta* | Q9FV46 |
| VvZDS | *Vitis vinifera* | I7EUP4 |
| ZmZDS | *Zea mays* | Q9ZTP4 |
| CnCRTI | *Cercospora nicotianae* | P48537 |
| EvCRTI | *Escherichia vulneris* | P22871 |
| NcCRTI | *Neurospora crassa* | P21334 |
| PaCRTI | *Pantoea ananas* | P21685 |
| PbCRTI | *Phycomyces blakesleeanus* | P54982 |
| SgCRTI | *Streptomyces griseus* | P54981 |
| AtCTRISO | *Arabidopsis thaliana* | Q9M9Y8 |
| CaCTRISO | *Capsicum annuum* | A0A1U8F6R6 |
| CsaCTRISO | *Cucumis sativus* | A0A0A0LBN7 |
| CsiCTRISO | *Citrus sinensis* | A0A067G5N7 |
| EsCRTISO | *Eutrema salsugineum* | V4KEQ2 |
| GmCTRISO | *Glycine max* | A0A0R0L3G5 |
| LcCTRISO | *Lycium chinense* | A0A140YIA2 |
| LsCTRISO | *Lactuca sativa* | A0A2J6KRW2 |
| NtCTRISO | *Nicotiana tabacum* | A0A1S3ZX27 |
| OsCTRISO | *Oryza sativa* | B8BL57 |
| SeCTRISO | *Synechococcus elongatus* | A0A3G6X1R9 |
| SlCTRISO | *Solanum lycopersicum* | Q8S4R4 |
| StCTRISO | *Solanum tuberosum* | M1CQH9 |
| VvCRTISO | *Vitis* *vinifera* | A0A438D0F6 |
