# Supplementary material for: Over-Expression of LcPDS, LcZDS, and LcCRTISO, Genes From Wolfberry for Carotenoid Biosynthesis, Enhanced Carotenoid Accumulation, and Salt Tolerance in Tobacco
Source: Front Plant Sci. 2020 Feb 26;11:119. doi: 10.3389/fpls.2020.00119 (PMC7054348; doi:10.3389/fpls.2020.00119)
Supplement: Supplementary file 12 [file Table_2.docx]

**Supplementary Table 2.** Primers used for vector construction in this study.

| **Gene** | **Primer sequences for vector construction (5'-3')** |
| --- | --- |
| *LcPDS* | F: ATCGGGATCCATGCCCCAAATTGGACTTGTT |
|  | R: ATCGGTCGACCGCAACTACGCTTGCTTCTGCCAA |
| *LcZDS* | F: ATCGGGATCCATGGCTACTTCTTCAGCTTATTTTTG |
|  | R: ATCGGTCGACGCAAGACTCAACTCATCAGATAGCGACA |
| *LcCRTISO* | F: ATCGGGATCCATGGGTACCTTGAATTTTATGTTTCC |
|  | R: ATCGGTCGACGCTGCTAGTGTCCTTAACCAAGCAAG |
