# Supplementary material for: Over-Expression of LcPDS, LcZDS, and LcCRTISO, Genes From Wolfberry for Carotenoid Biosynthesis, Enhanced Carotenoid Accumulation, and Salt Tolerance in Tobacco
Source: Front Plant Sci. 2020 Feb 26;11:119. doi: 10.3389/fpls.2020.00119 (PMC7054348; doi:10.3389/fpls.2020.00119)
Supplement: Supplementary file 13 [file Table_3.docx]

**Supplementary Table 3.** Primers used for qRT-PCR in this study.

| **Genes** | **Primer sequences for qRT-PCR (5'-3')** | **Accession number** |
| --- | --- | --- |
| *NtActin* | F: CCTGAGGTCCTTTTCCAACCA | XM_016658252.1 |
|  | R: GGATTCCGGCAGCTTCCATT |  |
| *NtGGPS* | F: CTGTCAATCGAGCCTTAGATGC | NM_001325671.1 |
|  | R: AAACCCGCTTGCCTTCG |  |
| *NtPSY* | F: TGTTGCTTTGTTGTGGGTTG | NM_001325140.1 |
|  | R: TTTGTCTCCCGCCTTTCA |  |
| *NtPDS* | F: AGTCAGACTAAACTCACGAATAAA | XM_016642616.1 |
|  | R: CTCCCACTAGCTTCTCCAA |  |
| *NtZDS* | F: GGGAGCTTGATTTCCGATTT | XM_016658599.1 |
|  | R: CCCGCACCACTGGACTAA |  |
| *NtCRTISO* | F: GCAGGACCAGATTCAGCG | NM_001325775.1 |
|  | R: GATGGATAAAGGAGGTAGAGCC |  |
| *Ntβ-LCY* | F: AAGAGCATTGAAGAGGACGAG | NM_001324787.1 |
|  | R: TGAGGGATGAACCAGACCAG |  |
| *Ntε-LCY* | F: GCTCTTGCTGCGGAGTC | NM_001325477.1 |
|  | R: GAATTGGATCGGCATCGT |  |
| *Ntβ-CHY* | F: CTTGGCAAATGGAGGGTG | NM_001326092.1 |
|  | R: AAGAAGAGCAATGGCTGGAA |  |
| *NtVDE* | F: CTGAAAGAGTGCAGGTTAGAGC | NM_001324805.1 |
|  | R: TGGAGACAGGCAACATTAGC |  |
| *NtZEP* | F: CAACAGCAGAAACTGAGCCAT | XM_016582459.1 |
|  | R: AACAATGACCAATAACTCCAGGC |  |
| *NtNSY* | F: AAGCCAAAGTAGGTGCCCAA | XM_016625496.1 |
|  | R: AACGGTAAGATGGAGAATGGCT |  |
| *NtAPX* | F: GGAAGAGTTGGGAGTGGTGG | NM_001324874.1 |
|  | R: ACAGCTGCCAAATTCAAATCCT |  |
| *NtP5CR* | F: AAGAGCGTGGCACAATCACT | XM_016610055.1 |
|  | R: AGCTTTTCTCGAGTTTTCTTCTGC |  |
| *NtPOD* | F: GGACAAATGGAGAGATTCGTTCAG | XM_016632447.1 |
|  | R: TGCTGACTTGGCCTACCAAC |  |
| *NtSOD* | F: TCCTCCCCAGGTTCGGAATA | XM_016631233.1 |
|  | R: TGCGCTACATTTGGCAAGAG |  |
| *LcPDS* | F: AGCAAGCGTAGTTTAGCCTAG | KJ143993 |
|  | R: AATGCTAATCTTCAAGTCGTC |  |
| *LcZDS* | F: CTGGTAAAGACCCATTCAGACC | KJ174516 |
|  | R: AAGACTCAACTCATCAGATAGCG |  |
| *LcCRTISO* | F: CATAGAAGATTGGGAGGGACT | KJ700839 |
|  | R: CAAGGTATCGCCTGTGGG |  |
