# Supplementary material for: Over-Expression of LcPDS, LcZDS, and LcCRTISO, Genes From Wolfberry for Carotenoid Biosynthesis, Enhanced Carotenoid Accumulation, and Salt Tolerance in Tobacco
Source: Front Plant Sci. 2020 Feb 26;11:119. doi: 10.3389/fpls.2020.00119 (PMC7054348; doi:10.3389/fpls.2020.00119)
Supplement: Supplementary file 14 [file Table_4.docx]

**Supplementary Table 4.** Information of carotenoids analyzed with HPLC

| **Peak Number** | **Chemical Name** | **CAS Number** | **Retention Time (second)** |
| --- | --- | --- | --- |
| 1 | neoxanthin | 14660-91-4 | 322.37 |
| 2 | violaxanthin | 126-29-4 | 369.71 |
| 3 | lutein | 127-40-2 | 525.26 |
| 4 | zeaxanthin | 144-68-3 | 570.35 |
| 5 | lycopene | 502-65-8 | 1118.2 |
| 6 | neurosporene | 502-64-7 | 1143.0 |
| 7 | ζ-carotene | 72746-33-9 | 1176.8 |
| 8 | β-carotene | 7235-40-7 | 1909.4 |
