# Supplementary material for: Over-Expression of LcPDS, LcZDS, and LcCRTISO, Genes From Wolfberry for Carotenoid Biosynthesis, Enhanced Carotenoid Accumulation, and Salt Tolerance in Tobacco
Source: Front Plant Sci. 2020 Feb 26;11:119. doi: 10.3389/fpls.2020.00119 (PMC7054348; doi:10.3389/fpls.2020.00119)
Supplement: Supplementary file 15 [file Table_5.docx]

**Supplementary Table 5.** Basic information of *LcPDS*, *LcZDS* and *LcCRTISO*.

| **Gene** | **Accession No.** | **Length of ORF (bp)** | **Number of amino acids (aa)** | **Molecular weight (Da)** | **pI of protein** |
| --- | --- | --- | --- | --- | --- |
| *LcPDS* | KJ143993 | 1749 | 582 | 64947.97 | 6.50 |
| *LcZDS* | KJ174516 | 1767 | 588 | 64686.19 | 8.32 |
| *LcCRTISO* | KJ700839 | 1815 | 604 | 66245.09 | 7.17 |
